# N-(2-Arylethyl)-2-methylprop-2-enamides as Versatile Reagents for Synthesis of Molecularly Imprinted Polymers

**DOI:** 10.3390/polym14132738

**Published:** 2022-07-04

**Authors:** Monika Sobiech, Dorota Maciejewska, Piotr Luliński

**Affiliations:** Department of Organic Chemistry, Faculty of Pharmacy, Medical University of Warsaw, Banacha 1, 02-097 Warsaw, Poland; monika.sobiech@wum.edu.pl (M.S.); dorota.maciejewska@wum.edu.pl (D.M.)

**Keywords:** *N*-acylation, phenethylamines, molecularly imprinted polymers, semi-covalent imprinting, tyramine

## Abstract

The paper describes the formation of six aromatic *N*-(2-arylethyl)-2-methylprop-2-enamides with various substituents in benzene ring, viz., 4-F, 4-Cl, 2,4-Cl_2_, 4-Br, 4-OMe, and 3,4-(OMe)_2_ from 2-arylethylamines and methacryloyl chloride in ethylene dichloride with high yields (46–94%). The structure of the compounds was confirmed by ^1^H NMR, ^13^C NMR, IR, and HR-MS. Those compounds were obtained to serve as functionalized templates for the fabrication of molecularly imprinted polymers followed by the hydrolysis of an amide linkage. In an exemplary experiment, the imprinted polymer was produced from N-(2-(4-bromophenyl)ethyl)-2-methylprop-2-enamide and divinylbenzene, acting as cross-linker. The hydrolysis of 2-(4-bromophenyl)ethyl residue proceeded and the characterization of material including SEM, EDS, ^13^C CP MAS NMR, and BET on various steps of preparation was carried out. The adsorption studies proved that there was a high affinity towards the target biomolecules tyramine and L-norepinephrine, with imprinting factors equal to 2.47 and 2.50, respectively, when compared to non-imprinted polymer synthesized from methacrylic acid and divinylbenzene only.

## 1. Introduction

Molecular-imprinting technology is engaged in searching for advanced selective materials with great potential for environmental, food, or biomedical analyses [1,2,3,4]. This technique forms polymers with desired selectivity towards a template, being a result of the interactions between the functional groups of template and monomer(s) prior to the polymerization process. The orientation of molecules is fixed through chemical cross-linking during the polymerization and then the removal of the template is undertaken to obtain a cavity in the molecularly imprinted polymer (MIP). Covalent or non-covalent imprinting strategies employ either chemical bonds or various weak interactions in the formation of template–monomer prepolymerization moieties [5].

The formation of stable prepolymerization structures is a critical step during the imprinting process. The use of a template with covalently bound polymerizable units (a functionalized template) prior to the polymerization resulted in the formation of well-defined binding sites in the polymer matrix. Chemical cleavage is required at the final stage of the process. The fabrication of MIPs, applying the monomer covalently bound to the template, is followed by different rebinding/adsorption approaches, e.g., the rebinding of the target analyte to the polymer matrix by covalent bonds [6] or adsorption of the target analyte via non-covalent intermolecular interactions [7].

The advancement of the use of a functionalized template resulted in a more homogeneous population of binding sites in the resultant MIP and greater binding-site integrity when compared to the MIP synthesized with a non-covalent strategy [8,9,10]. Hashim and co-workers [8] compared the covalent and non-covalent imprinting strategies for the synthesis of stigmasterol imprinted polymers. In the non-covalent imprinting strategy, stigmasterol was selected as the template and methacrylic acid or 4-vinylpyridine were used as the functional monomers to form different MIPs. In the covalent imprinting strategy, stigmasteryl-3-*O*-methacrylate was synthesized prior to its application as the functionalized template. It was found that the non-covalent imprinting method showed insufficient binding affinity and low selectivity towards stigmasterol. In contrast, the application of covalent imprinting in the formation of MIP followed by the chemical cleavage of ester bonds resulted with highly selective imprinted polymer. Similar results were described by Tang and co-workers [9]. Here, the simultaneous reaction of *N*- and *O*-acylation of ractopamine was provided to obtain a novel functionalized template. The results were compared with previously published studies of non-covalent imprinting of ractopamine [10]. It was found that MIPs obtained by the covalent imprinting strategy possessed significantly higher binding capacity and selectivity. The homogeneous population of binding sites towards ractopamine in the covalently produced MIP was confirmed by isotherm equilibrium-binding experiments [9], providing a substantial difference between it and the heterogeneous population of binding sites observed in the non-covalently formed MIP [10]. More recently, the covalent approach was used to synthesize more advanced and selective materials integrated with metal-organic frameworks [11], magnetic cores [12], surfaces of microtiter plates [13], or dendritic fibrous silica [14].

The current investigations of our group aim to explore the ability of MIPs produced by the covalent imprinting strategy to selectively recognize biogenic amines with 2-phenylethylamine system (phenethylamine system) [15,16]. The group of compounds that contain a backbone of phenethylamine play a very important role in the human nervous system. These molecules act as neurotransmitters and neuromodulators or psychotropic agents, causing neurological disorders related to mood, emotion, attention, and cognition when their levels are irregular or produce hallucinations, illusions, or mental disorders when prolonged or overdosed [17,18]. Moreover, the presence of these compounds, predominantly at low levels in highly complex samples, hampers their analysis. Thus, investigations of selective materials with satisfactory clean-up capabilities for the separation of phenethylamines are completely justified. Here, advanced polymeric materials could improve the detection of the above-mentioned biomolecules as well as contribute to explaining some aspects of neurological diseases or drug addiction.

The aim of the study was to synthesize and characterize various N-(2-arylethyl)-2-methylprop-2-enamides. The potential of those reagents for the fabrication of specific MIPs was proved in an exemplary synthesis of a molecularly imprinted polymer, using one of synthesized compounds as a functionalized template, followed by the analysis and characterization of the resultant material. In this paper, the synthesis of compounds that possess a template fragment of phenethylamine covalently bound with a polymerizable unit is presented, as well as the primary verification of their ability to synthesize an imprinted sorbent for the analysis of phenethylamines.

## 2. Materials and Methods

### 2.1. Materials

#### 2.1.1. Reagents

The following are the relevant ethylamines: 2-(4-fluorophenyl)ethylamine (**1a**), 2-(4-chlorophenyl)ethylamine (**1b**), 2-(2,4-dichlorophenyl)ethylamine (**1c**), 2-(4-bromophenyl)ethylamine (**1d**), 2-(4-methoxyphenyl)ethylamine (**1e**), 2-(3,4-dimethoxyphenyl)ethylamine (**1f**). The target analytes are as follows: tyramine; L-norepinephrine or 3,4-dihydroxyphenylacetic acid; and methacrylic acid, methacryloyl chloride, divinylbenzene were sourced from Sigma-Aldrich (Steinheim, Germany). The polymerization reaction initiator, 1,1′-azobiscyclohexanecarbonitrile, was from Merck (Darmstadt, Germany). The relevant solvents (ethylene dichloride, hexane, petroleum ether, toluene, methanol, hydrochloric acid (36%), triethylamine, and salts used in the synthesis and the post-polymerization treatment) were delivered from POCh (Gliwice, Poland). Ultra-pure water delivered from a Milli-Q purification system (Millipore, France) was used to prepare water solutions.

#### 2.1.2. Synthesis of N-(2-arylethyl)-2-methylprop-2-enamides

**Scheme 1:**
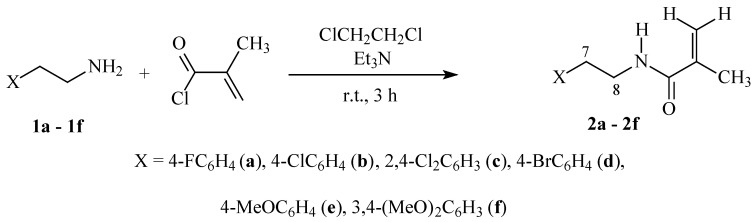
Synthesis of *N*-(2-arylethyl)-2-methylprop-2-enamides.

The selected amines **1a**–**1f** (10 mmol) were added to ethylene dichloride (30 mL) and triethylamine (1.67 mL, 12 mmol) under stirring. Subsequently, methacryloyl chloride (1.18 mL, 12 mmol, 20% excess) was slowly added dropwise to the reaction mixture for 15 min. A white precipitate of triethylammonium chloride was formed and the reaction mixture was stirred for 3 h in room temperature. The precipitated salt was filtered off and the remaining organic layer was washed with saturated NaHCO_3_ and water, prior to drying over anhydrous MgSO_4_. Following this, the layer was evaporated under vacuum yielding crude solid products which were recrystallized from hexane or petroleum ether to obtain pure products **2a**–**2f** (Scheme 1).

#### 2.1.3. Preparation of Polymers

The synthesis of the imprinted material, coded MIP**_ft_**, was carried out. The functionalized template (ft) was used to form an imprinted material. A functionalized template could be defined as the template that possesses one or more polymerizable units that are attached by covalent bonds to form a template–monomer structure by a chemical step independent of polymer formation. In the synthesis of MIP**_ft_**, the compound **2d** (175.39 mg; 0.8 mmol) was added to toluene (2.056 mL). In the synthesis of NIP, methacrylic acid (69 mg; 0.8 mmol) was added to the same volume of toluene. Following this, divinylbenzene (570 µL; 4 mmol) and 1,1′-azobiscyclohexanecarbonitrile (10 mg) were added to the prepolymerization mixture prior to purging the mixture with nitrogen for 3–5 min. The polymerization process was carried out in 88–92 °C for 24 h. Subsequently, the bulk rigid polymers were ground in a mortar with a pestle and wet-sieved into particles below 45 μm diameter prior to discarding the fine particles by repeated decantation in acetone. Following this, the imprinted particles were treated under reflux with 1 mol L^−1^ hydrochloric acid for 3 h (50 mL) in order to hydrolyze the amide linkage and to remove 4-bromophenylethylamine residue. For comparison, NIP was treated in the same way. Finally, the particles were extensively washed with methanol and were dried prior to the analysis.

#### 2.1.4. Binding Studies

Empty 1 mL solid-phase extraction cartridges were filled with 25 mg of MIP**_ft_** or NIP and secured by fiberglass frits. The polymers were then conditioned with methanol–water (85:15 *v*/*v*, 1 mL), and loaded with a standard solution of **1d** (conc. 50 μmol L^−1^, methanol–water, 85:15 *v*/*v*, 5 mL) or tyramine (conc. 20 μmol L^−1^, methanol–water, 85:15 *v*/*v*, 5 mL). The unbound amounts of **1d** or tyramine were determined with reference to their respective calibration lines and UV spectroscopy was used for detection. The bound amounts were calculated by subtracting the unbound amount of **1d** or tyramine from initial amount of **1d** or tyramine, respectively. For selectivity tests, L-norepinephrine (conc. 20 μmol L^−1^) or 3,4-dihydroxyphenylacetic acid (conc. 20 μmol L^−1^) were used and the analysis was carried out in the same way as described above. The binding capacities and specificity of materials were evaluated [5,19]. The binding capacities (*B*, μmol g^−1^) were calculated according to Equation (1):

(1) $$B=\frac{\left(C_{i}-C_{f}\right)V}{M}$$

where *V* represents the volume of portion (L) in each loading step, *C_i_* represents the initial analyte concentration (µmol L^−1^), *C_f_* represents the analyte concentration in solution after adsorption (µmol L^−1^), and *M* is the mass of polymer particles (g). The binding capacities of MIP**_ft_** and NIP were compared by the determination of the imprinting factor (IF) calculated according to Equation (2):

(2) $$\mathrm{IF}=\frac{B_{MIP}}{B_{NIP}}$$

For isotherm analysis, equilibrium-binding experiments were applied. The polypropylene tubes were filled with 10 mg MIP**_ft_** or NIP. Next, a volume of 50 mL of methanol–water (85:15 *v/v*) standard solution of tyramine was added (concentration range between 10–200 μmol L^−1^). The tubes were sealed and oscillated by a shaker at room temperature for 24 h prior to centrifugation for 10 min. The aliquots were then used to analyze the unbound amount of tyramine. The amount of tyramine bound to the polymer was calculated according to Equation (1). The detailed analyses of adsorption on MIP**_ft_** and NIP were provided using Lineweaver–Burk model [20] represented by Equation (3):

(3) $$\frac{1}{B}=\frac{1}{B_{\max}}+\frac{1}{K_{L}B_{\max}F}$$

where *B*_max_ is the maximum binding capacity and *K*_L_ is the equation constant. The Freundlich model [21] represented by Equation (4) was also employed:

*B* = *aF^m^* (4)

where *B* is the adsorbed amount of analyte, *F* is the unbound amount of analyte, *a* is the measure of the capacity (*B*_max_) and *m* is a heterogeneity index.

#### 2.1.5. Physicochemical Characterization

The ^1^H NMR, ^13^C NMR spectra of **2a**–**2e** and the ^13^C CP/MAS NMR spectrum of MIP_ft_ in solid state were recorded with a Bruker Avance DMX 400 spectrometer (Bruker, Germany) at the Faculty of Pharmacy, Medical University of Warsaw, Poland. For the ^13^C CP/MAS NMR, a powdered sample was contained in 4 mm ZrO_2_ rotors and was spun at 8 kHz. The 90° pulse length was 2.15 µs. A contact time of 4 ms and a repetition time of 10 s were used for the accumulation of 1900 scans. The chemical shifts, δ ppm, were referenced to tetramethylsilane.

Spectroscopic analyses were carried out using a UV-1605PC spectrophotometer (Shimadzu, Germany). The calibration lines as a function of absorbance (y) versus concentration (x) were constructed at λ_max_ of the analyzed compounds. Each point was measured in triplicate. The linearities of calibration lines were good, with correlation coefficients r^2^ > 0.997.

Scanning electron microscopy (SEM) and energy-dispersive X-ray spectrophotometry (EDS) for MIP**_ft_** were studied on Merlin FE-SEM (Zeiss, Germany) combined with an EDS X-ray detector (Brucker, Germany). The samples were Au/Pd sputter-coated (SEM) or carbon-coated (EDS) before analysis. The analyses were performed at the Faculty of Chemistry, University of Warsaw, Poland.

The porosity data for MIP**_ft_** were determined using the adsorption isotherm of N_2_ at 77 K (BET) on an ASAP 2420 system (Micromeritics Inc., Norcross, GA, USA) at the Faculty of Chemistry, Maria Curie-Skłodowska University, Lublin, Poland.

## 3. Results and Discussion

### 3.1. Synthesis and Identification of N-(2-arylethyl)-2-methylprop-2-enamides

In order to obtain reagents for the fabrication of imprinted polymers, the procedure for the synthesis of six *N*-(2-arylethyl)-2-methylprop-2-enamides from *N*-(2-arylethyl)amines with various substituents (methoxy or halogens) in benzene ring was described (Scheme 1). The respective *N*-(2-arylethyl)-2-methylprop-2-enamides were isolated with high yields (Table 1) and their chemical structures were determined by high-resolution mass spectrometry, IR and NMR analyses.

The conversion of phenethylamine to phenethylamide is usually a straightforward matter, involving reactions with acyl chloride [22] or acetyl anhydride [23]. Weiner and co-workers [22] used methacryloyl chloride as acylating agent to obtain *N*-(2-(4-hydroxyphenyl)ethyl)-2-methylprop-2-enamide. The corresponding *N*-acylations were made in ethyl ether or in benzene, but are inconvenient because of the low boiling point and high toxicity, respectively. Rathelot and co-workers [23] used the *N*-acylation reaction of phenethylamine to obtain 2-phenylethylacetamide, a substrate in the multistep synthesis of novel antimalarial drugs. In the above-mentioned reaction, acetic anhydride was applied as the *N*-acylation reagent.

Here, ethylene dichloride was used as a solvent and methacryloyl chloride as acylating agents to obtain derivatives of *N*-(2-arylethyl)-2-methylprop-2-enamides. The presence of triethylamine provides a satisfactory method of neutralizing the hydrogen halide for amide synthesis. The reaction was finished within 3 h at room temperature. A series of 2-arylethylamines with various substituents in benzene ring, viz., 4-F, 4-Cl, 2,4-Cl_2_, 4-Br, 4-OMe, 3,4-(OMe)_2_, **1a**–**1f** were then used as substrates (Table 1). These amines were selected because of their structural similarity to the biogenic amines or synthetic psychoactive substances used in designer drugs. The conversion of substrates was monitored by TLC. The formation of any other products apart from the main products of the *N*-acylation reaction was not observed. The structures of compounds **2a**–**2f** were confirmed by spectroscopy (Table 2).

It has to be underlined that the screening of chemical databases revealed only the presence of *N*-(2-(4-iodophenyl)ethyl)-2-methylprop-2-enamide [24] and *N*-(2-(2-bromophenyl)ethyl)-2-methylprop-2-enamide [25,26].

Hence, it could be assumed that the *N*-acylation reaction of phenethylamines with methacryloyl chloride is effective for phenethylamines with the strong electron-donating group –OCH_3_ or with the halogens F, Cl, or Br in a benzene ring.

### 3.2. Preparation of Polymer

In an antecedent paper, Weiner and co-workers [22] described the effect of the attachments of various phenethylamines, viz., phenethylamine, tyramine, ephedrine (2-(methylamino)-1-phenylpropan-1-ol), and amphetamine into synthetic or natural polymers by an amide or carbamate linkage as a method for increasing their duration of action. However, the imprinting technique was not studied in the above-mentioned paper.

**Figure 1:**
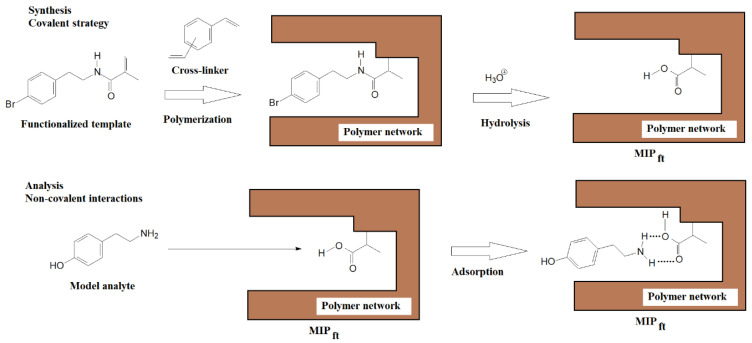
Schematic idea of the synthesis, employing a covalent strategy for the imprinting process and an adsorption process on the resultant imprinted polymer that is based on non-covalent interactions of the target analytes.

Here, we employed the covalent imprinting approach to synthesize the polymers but the adsorption process on imprinted material was entirely non-covalent in nature [27]. Thus, so-called ‘semi-covalent imprinting’ was applied. In order to confirm the structure, to analyze the morphology, and to prove the selectivity of the resultant imprinted polymer, the synthesis of MIP**_ft_** from *N*-(2-(4-bromophenyl)ethyl)-2-methylprop-2-enamide, **2d**, was carried out in the presence of divinylbenzene (cross-linker) in toluene (porogen). Radical thermal polymerization was applied to obtain bulk material. The schematic idea of the synthesis, employing a covalent strategy for the imprinting process and an adsorption process on the resultant imprinted polymer that is based on non-covalent interactions of the target analytes, is presented in Figure 1. The NIP was prepared with the employment of methacrylic acid as the functional monomer and divinylbenzene as the cross-linker, omitting the addition of any template, and it was fabricated to compare sorption behavior**.**

The amide linkage, present in the MIP**_ft_**, was hydrolyzed prior to the removal of 2-(4-bromophenyl)ethylamine residue in order to form specific cavities in the imprinted polymer. The hydrolysis was carried out in 1 mol L^−1^ hydrochloric acid under reflux. The hydrolysis lasted up to 3 h.

### 3.3. Characterization of Material

#### 3.3.1. Adsorption Behavior

The binding capacities were determined for MIP**_ft_** as well as for NIP in one μmol of **1d** for one gram of polymer particles. The selectivity of the imprinted polymer (imprinting factor, IF) was expressed as the ratio of the amount of **1d** bound to MIP**_ft_** in comparison to NIP.

The binding capacities of **1d** and IF were as follows: MIP**_ft_**: 8.09 ± 0.08 μmol g^−1^, NIP: 4.59 ± 0.04 μmol g^−1^, IF = 1.76. These results proved that the functionalized template **2d** could be used as reagent for covalent imprinting and the resulting MIP**_ft_** was possessed of selectivity when compared to NIP.

**Figure 2:**
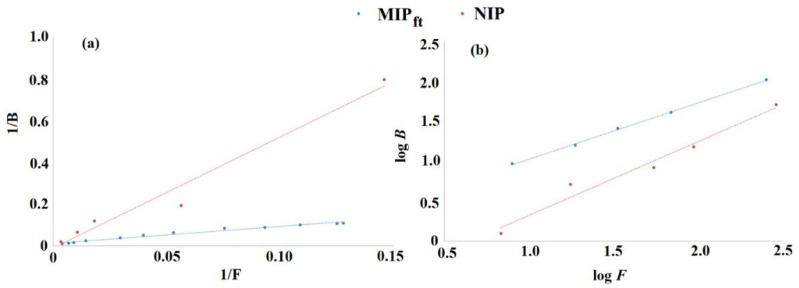
Lineweaver–Burk (**a**) and Freundlich (**b**) models for tyramine adsorption on MIP**_ft_** and NIP.

Sorption behavior is responsible for the effective separation of target analytes on MIPs. In order to prove that the novel imprinted material possessed an affinity towards biogenic amines with a phenethylamine system, the adsorption of tyramine on MIP**_ft_** was examined. The binding capacities of tyramine on MIP**_ft_** and on NIP were as follows: 2.74 ± 0.03 μmol g^−1^ and 1.11 ± 0.01 μmol g^−1^, respectively (IF = 2.47). In order to analyze adsorption data, the Langmuir isotherm was applied [28]. Here, various linearized modifications of the model could be used to determine the most proper fit [29]. It was found that the Lineweaver–Burk modification represented by Equation (3) was characterized by the highest regression coefficients for analyzed data with values of *r*^2^ = 0.974 and *r*^2^ = 0.971 for MIP**_ft_** and NIP, respectively. Moreover, to reveal the homogeneity of MIP**_ft_**, the Freundlich model described by Equation (4) was applied. That model fits well to MIP adsorption data in the low-concentration regions and allows for the surface-homogeneity determination of the material tested. The straight line of log *B* versus log *F* is the evidence that adsorption can be described by the Freundlich equation. The correlation coefficients, *r*^2^, for MIP**_ft_** and NIP were equal to 0.999 and 0.972, respectively, and the estimated values of *m* were equal to 0.70 and 0.93 for MIP**_ft_** and NIP, respectively (Figure 2).

It was found that MIP**_ft_** provided affinity towards tyramine. In order to analyze the selectivity, two other biomolecules were used for studies, viz., L-norepinephrine and 3,4-dihydroxyphenylacetic acid. L-norepinephrine possesses a phenethylamine system. In contrast, 3,4-dihydroxyphenylacetic acid (a metabolite in the dopamine system) does not possess a phenethylamine system. The binding capacities for MIP**_ft_** and NIP were as follows: for L-norepinephrine, 14.5 ± 1.4 μmol g^−1^ and 5.80 ± 0.59 μmol g^−1^, respectively (IF = 2.50); and for 3,4-dihydroxyphenylacetic acid, 0.579 ± 0.070 μmol g^−1^ and 0.549 ± 0.060 μmol g^−1^, respectively (IF = 1.06). The results show the selectivity of MIP**_ft_** to L-norepinephrine, a biomolecule with a phenethylamine system, and its lack of selectivity to 3,4-dihydroxyphenylacetic acid, a biomolecule that not possess a phenethylamine system. The higher binding capacity of L-norepinephrine when compared to the binding capacity of tyramine could be explained by the presence of two strong electron-donating hydroxy groups in positions 3 and 4 of the aromatic ring and one hydroxy group in the aliphatic ethylamine chain, enhancing strongly the basicity of L-norepinephrine. Apart from being an exemplary experiment, the results are very promising for the possible application of such reagents in the preparation of sorbents for biomedical purposes.

#### 3.3.2. Morphology Characterization

**Figure 3:**
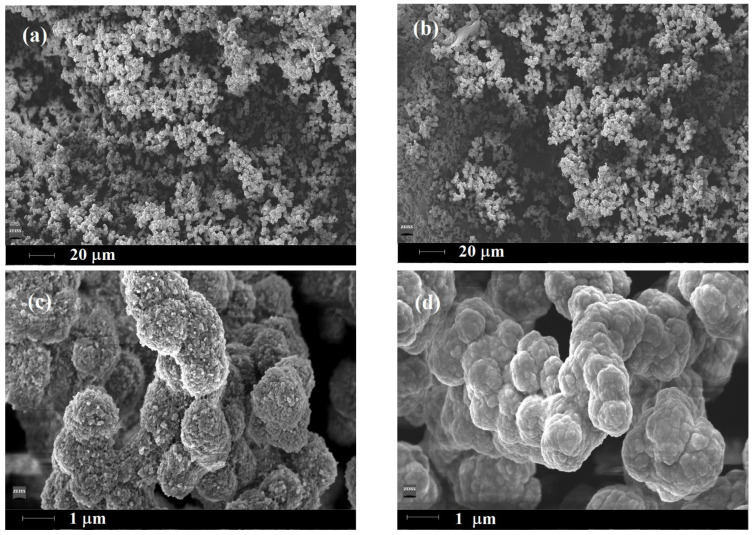

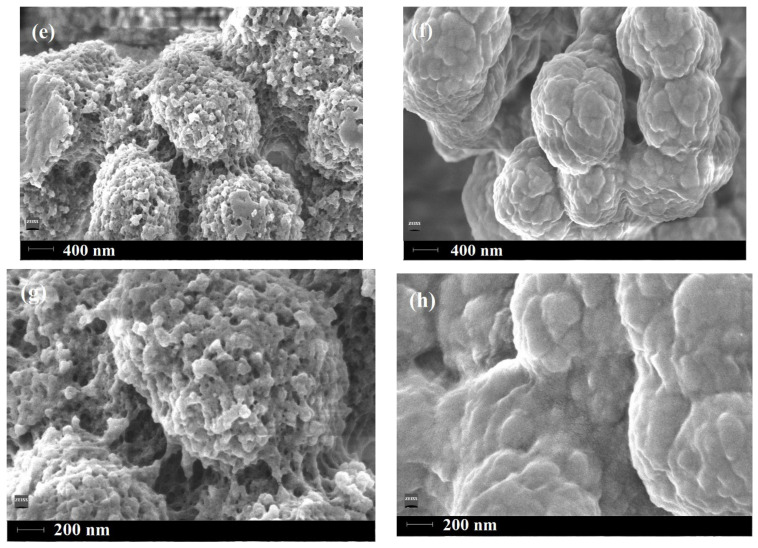
SEM micrographs of MIP**_ft_** (**a**,**c**,**e**,**g**) and NIP (**b**,**d**,**f**,**h**).

In order to provide morphological characterization, scanning electron microscopy was employed and the surface of MIP**_ft_** was analyzed. Figure 3 presents a micrograph of MIP**_ft_** after the hydrolysis process. The particles possessed the morphology of the bulk materials that were composed from spherical entities agglomerated into bigger forms of 10–20 µm. The diameter of single entity varied from 500 nm to 2 µm and was similar for MIP**_ft_** and NIP (Figure 3a–d). However, further magnification revealed substantial difference between MIP**_ft_** and NIP (Figure 3e–h). The MIP**_ft_** was characterized by significant surface extension with numerous macropores clearly detected on the particle’s surface. On the contrary, the NIP possessed a smoother surface. The difference could be related to the presence of the functionalized template in the prepolymerization mixture.

#### 3.3.3. Structural Evaluation

**Figure 4:**
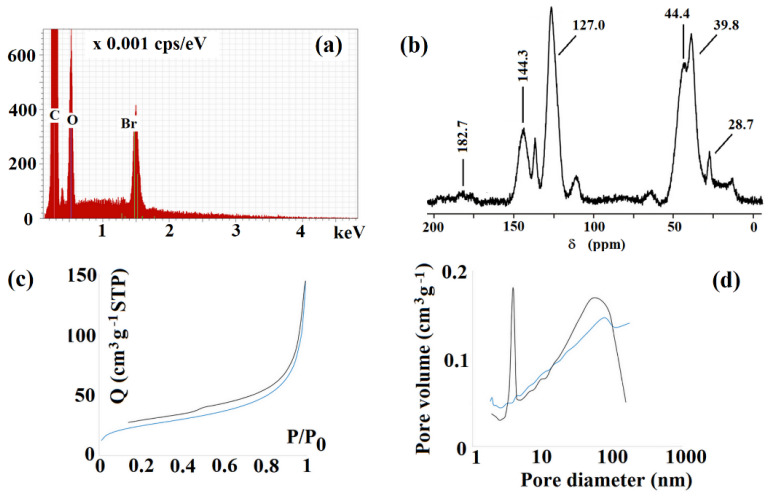
EDS spectrum of MIP**_ft_** before hydrolysis of the amide linkage (**a**), ^13^C CP MAS NMR spectrum for MIP**_ft_** (**b**), nitrogen-sorption hysteresis (**c**) and pore-size distributions (**d**) for MIP**_ft_** after hydrolysis of amide linkage.

In order to confirm the structure of the resultant polymers, EDS was used to prove that the functionalized template was polymerized (Figure 4a). The MIP**_ft_** was prepared in order to confirm that the functionalized template was built up into the polymer matrix because the presence of bromine atoms in the structure of **2d** allowed us to detect heteroatom during the analysis of the materials. It has to be underlined that the analysis of the polymer, viz., MIP**_ft_**, was carried out omitting the process of the hydrolysis of the amide linkage. The atoms of bromine were detected in the MIP**_ft_** structure in the region of 1.50 keV.

^13^C CP/MAS NMR spectroscopy was then applied. This is a versatile tool to confirm the composition of polymer materials. For the purpose of our analysis, the MIP**_ft_** was post-treated to remove 2-(4-bromophenyl)ethylamine residue from the polymer matrix (Figure 4b). In the ^13^C CP/MAS NMR spectrum of MIP**_ft_**, strong resonances in the aromatic region, representing quaternary benzene C atoms at 137.2 and 144.3 ppm, could be seen. The tertiary –CH atoms at 127.0 ppm originated from the cross-linker. In the aliphatic region, various methyl groups were represented by broad peaks located between 15 and 30 ppm with a narrower sharp peak at 28.7 ppm. Methylene groups in C–CH_2_–C were found in approximately 44.4 ppm and methylene groups in Φ–CH–C in 39.8 ppm. Carboxyl group, –COOH, atoms were represented by broad resonances in the region of 177.1–182.7 ppm. Low intensity resonance at 111.7 ppm could originate from unreacted double bonds in Φ–(CH=CH_2_)_2_.

#### 3.3.4. Porosity Data

Finally, the nitrogen-adsorption isotherm (Brunauer–Emmett–Teller) for MIP**_ft_** was analyzed. As it can be seen (Figure 4c) the material revealed physisorption isotherms of type IV with a hysteresis loop. The shape of the hysteresis loop is related to the specific pore structure. Here, type H3 loops characterized MIP**_ft_**, indicating the slit-shaped structure of its pores. However, the deformation of the desorption-hysteresis line of MIP**_ft_** in the region of 0.50 P/P_o_ could be related to the expulsion of adsorbate from larger-volume mesopores through narrower pore necks. Thus, a more complicated pore system could exist in MIP**_ft_**. The total specific surface area of MIP**_ft_** was equal to 89.88 m^2^ g^−1^ and the external surface area was equal to 77.99 m^2^ g^−1^. The plots of pore volume versus diameter for MIP**_ft_** showed a peak for a pore diameter of 56 nm (Figure 4d).

## 4. Conclusions

In conclusion, it should be emphasized that a series of compounds, *N*-(2-arylethyl)-2-methylprop-2-enamides, were obtained with the synthetic procedure presented here with high yields. These compounds possessed fragments of a template covalently bound to polymerizable units and could be used as reagents for the covalent imprinting of polymers. In the control experiment, one of synthesized compounds was used to produce an imprinted polymer (MIP**_ft_**). Binding-capacity analysis revealed that a molecular imprinting process took place and the polymer, MIP**_ft_**, possessed selectivity towards biomolecules of tyramine and L-norepinephrine.

## Figures and Tables

**Table 1:** Amines (**1a**–**1f**) used in synthesis followed by yields and melting points of respective *N*-(2-arylethyl)-2-methylprop-2-enamides (**2a**–**2f**).

Amine	Substituents	Product	Yield (%)	M.p. (°C)
**1a**	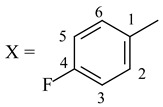	**2a**	90	74–75
**1b**	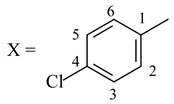	**2b**	81	103–104
**1c**	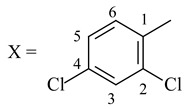	**2c**	92	104–105
**1d**	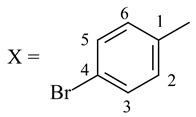	**2d**	46	114–115
**1e**	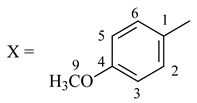	**2e**	96	74–75
**1f**	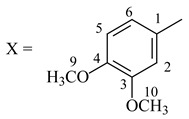	**2f**	63	63–64

**Table 2:** Spectral data of synthesized compounds.

	Compound, ^1^H, ^13^C NMR, IR and MS Data
**2a**	*N-(2-(4-fluorophenyl)ethyl)-2-methylprop-2-enamide*: ^1^H NMR (300 MHz, CDCl_3_) *δ* (ppm) = 7.19-7.13 (m, 2H, H2, H6), 7.04-6.96 (m, 2H, H3, H5), 5.78 (s, 1H, NH), 5.60 (bt, 1H, H3′a), 5.29 (quint, 1H, H3′b), 3.55 (m, 2H, H8), 2.83 (t, 2H, H7, *J* = 6.9 Hz), 1.92 (dd, 3 H, H4′, *J*_1_ = 1.2 Hz, *J*_2_ = 1.5 Hz); ^13^C NMR (75 MHz, CDCl_3_) *δ* (ppm) = 168.86 (C1′), 162.17 (C4), 140.56 (C2′), 135.05 (C1), 130.66 (C2,C6), 119.84 (C3′), 115.94 (C3,C5), 41.32 (C8), 35.31 (C7), 19.09 (C4′); IR (cm^−1^) 3342 (NH), 1656 (C=O), 1615 (C=C); MS (*m/z*, 70 eV) 207.11 [M^+^] (calc. 207.20).
**2b**	*N-(2-(4-chlorophenyl)ethyl)-2-methylprop-2-enamide*: ^1^H NMR (300 MHz, CDCl_3_) *δ* (ppm) = 7.31-7.26 (m, 2H, H3,H5), 7.15-7.11 (m, 2H, H2,H6), 5.81 (bs, 1H, NH), 5.60 (bt, 1H, H3′a), 5.30 (quint, 1H, H3′b, *J* = 1.5 Hz), 3.54 (q, 2H, H8, *J* = 6.9 Hz), 2.83 (t, 2H, H7, *J* = 6.9 Hz), 1.92 (dd, 3H, H4′, *J*_1_ = 1.2 Hz, *J*_2_ = 1.5 Hz); ^13^C NMR (75 MHz, CDCl_3_) *δ* (ppm) = 168.87 (C1′), 140.51 (C2′), 137.86 (C1), 132.87 (C4), 130.59 (C3,C5), 129.24 (C2,C6), 119.89 (C3′), 41.15 (C8), 35.48 (C7), 19.09 (C4′); IR (cm^−1^) 3316 (NH), 1652 (C=O), 1615 (C=C); MS (*m/z*, 70 eV) 223.08 [M^+^] (calc. 223.66).
**2c**	*N-(2-(2,4-dichlorophenyl)ethyl)-2-methylprop-2-enamide*: ^1^H NMR (300 MHz, CDCl_3_) *δ* (ppm) = 7.38 (d, 1H, H3, *J* = 1.8 Hz), 7.19 (m, 1H, H5, *J*_1_ = 8.1 Hz, *J*_2_ = 1.8 Hz), 7.16 (m, 1H, H6, *J* = 8.1 Hz), 5.96 (bs, 1H, NH), 5.63 (bs, 1H, H3′a), 5.30 (quint, 1H, H3′b, *J* = 1.5 Hz), 3.56 (q, 2H, H8, *J* = 7 Hz), 2.98 (t, 2H, H7, *J* = 7 Hz), 1.93 (dd, 3H, H4′, *J*_1_ = 1.2 Hz, *J*_2_ = 1.5 Hz); ^13^C NMR (75 MHz, CDCl_3_) *δ* (ppm) = 168.95 (C1′), 140.39 (C2′), 135.73 (C1), 135.19 (C2), 133.52 (C4), 132.25 (C6), 129.85 (C3), 127.71 (C5), 119.97 (C3′), 39.68 (C8), 33.19 (C7), 19.08 (C4′); IR (cm^−1^) 3299 (NH), 1654 (C=O), 1616 (C=C); MS (*m/z*, 70 eV) 257.09 [M^+^] (calc. 258.10).
**2d**	*N-(2-(4-bromophenyl)ethyl)-2-methylprop-2-enamide*: ^1^H NMR (300 MHz, CDCl_3_) *δ* (ppm) = 7.46-7.41 (m, 2H, H3,H5), 7.10-7.06 (m, 2H, H2,H6), 5.79 (bs, 1H, NH), 5.60 (bt, 1H, H3′a, *J* = 1.5 Hz), 5.30 (bsextet, 1H, H3′b, *J* = 1.5 Hz), 3.54 (bq, 2H, H8, *J* = 7 Hz), 2.82 (t, 2H, H7, *J* = 7 Hz), 1.92 (dd, 3H, H4′, *J*_1_= 0.9 Hz, *J*_2_ = 1.5 Hz); ^13^C NMR (75 MHz, CDCl_3_) *δ* (ppm) = 168.88 (C1′), 140.51 (C2′), 138.39 (C1), 132.21 (C3,C5), 131.00 (C2,C6), 120.90 (C4), 119.90 (C3′), 41.10 (C8), 35.55 (C7), 19.10 (C4′); IR (cm^−1^) 3318 (NH), 1653 (C=O), 1611 (C=C); MS (*m/z*, 70 eV) 267.03 [M^+^] (calc. 268.12).
**2e**	*N-(2-(4-methoxyphenyl)ethyl)-2-methylprop-2-enamide*: ^1^H NMR (300 MHz, CDCl_3_) *δ* (ppm) = 7.12 (m, 2H, H2,H6), 6.86 (m, 2H, H3,H5), 5.84 (bs, 1H, NH), 5.60 (bt, 1H, H3′a, *J* = 0.9 Hz), 5.28 (quint, 1H, H3′b, *J* = 1.5 Hz), 3.79 (s, 3H, H9), 3.53 (m, 2H, H8), 2.79 (t, 2H, H7, *J* = 6.9 Hz), 1.92 (dd, 3H, H4′, *J*_1_ = 0.9 Hz, *J*_2_ = 1.5 Hz); ^13^C NMR (75 MHz, CDCl_3_) *δ* (ppm) = 168.79 (C1′), 158.76 (C4), 140.59 (C2′), 131.33 (C1), 130.16 (C2,C6), 119.71 (C3′), 114.52 (C3,C5), 55.71 (C9), 41.39 (C8), 35.14 (C7), 19.07 (C4′); IR (cm^−1^) 3303 (NH), 1652 (C=O), 1615 (C=C); MS (*m/z*, 70 eV) 219.13 [M^+^] (calc. 219.24).
**2f**	*N-(2-(3,4-dimethoxyphenyl)ethyl)-2-methylprop-2-enamide*: ^1^H NMR (300 MHz, CDCl_3_) *δ* (ppm) = 6.82 (d, 1H, H5, *J* = 8.4 Hz), 6.74 (dd, 1H, H6, *J*_1_ = 1.8 Hz, *J*_2_ = 8.4 Hz), 6.72 (d, 1H, H2, *J* = 1.8 Hz), 5.81 (s, 1H, NH), 5.61 (bt, 1H, H3′a), 5.29 (quint, 1H, H3′b, *J* = 1.5 Hz), 3.87 (s, 6H, H9,H10), 3.55 (q, 2H, H8, *J* = 6.9 Hz), 2.81 (t, 2 H, H7, *J* = 6.9 Hz), 1.92 (bt, 3H, H4′); ^13^C NMR (75 MHz, CDCl_3_) *δ* (ppm) = 168.81 (C1′), 149.56 (C3), 148.21 (C4), 140.61 (C2′), 131.90 (C1), 121.13 (C6), 119.78 (C3′), 112.41 (C2), 111.86 (C5), 56.41 (C10), 56.32 (C9), 41.36 (C8), 35.64 (C7), 19.10 (C4′); IR (cm^−1^) 3320 (NH), 1650 (C=O), 1618 (C=C); MS (*m/z*, 70 eV) 249.14 [M^+^] (calc. 249.27).

## Data Availability

Not applicable.
